# 14-3-3 and Smad2/3 are crucial mediators of atypical-PKCs: Implications for neuroblastoma progression

**DOI:** 10.3389/fonc.2023.1051516

**Published:** 2023-01-20

**Authors:** S. Breedy, W.S. Ratnayake, L. Lajmi, R. Hill, M. Acevedo-Duncan

**Affiliations:** ^1^ Department of Chemistry, University of South Florida, Tampa, FL, United States; ^2^ Department of Cell Biology, Microbiology and Molecular Biology, University of South Florida, Tampa, FL, United States

**Keywords:** PKC-iota, PKC-zeta, 14-3-3, Smad2/3, neuroblastoma, targeted therapy

## Abstract

Neuroblastoma (NB) is a cancer that develops in the neuroblasts. It is the most common cancer in children under the age of 1 year, accounting for approximately 6% of all cancers. The prognosis of NB is linked to both age and degree of cell differentiation. This results in a range of survival rates for patients, with outcomes ranging from recurrence and mortality to high survival rates and tumor regression. Our previous work indicated that PKC-ι promotes cell proliferation in NB cells through the PKC-ι/Cdk7/Cdk2 cascade. We report on two atypical protein kinase inhibitors as potential therapeutic candidates against BE(2)-C and BE(2)-M17 cells: a PKC-ι-specific 5-amino-1-2,3-dihydroxy-4-(methylcyclopentyl)-1H-imidazole-4-carboxamide and a PKC-ζ specific 8-hydroxy-1,3,6-naphthalenetrisulfonic acid. Both compounds induced apoptosis and retarded the epithelial-mesenchymal transition (EMT) of NB cells. Proteins 14-3-3 and Smad2/3 acted as central regulators of aPKC-driven progression in BE(2)-C and BE(2)-M17 cells in relation to the Akt1/NF-κB and TGF-β pathways. Data indicates that aPKCs upregulate Akt1/NF-κB and TGF-β pathways in NB cells through an association with 14-3-3 and Smad2/3 that can be diminished by aPKC inhibitors. In summary, both inhibitors appear to be promising potential neuroblastoma therapeutics and merit further research.

## Introduction

Neuroblastoma (NB) is a pediatric cancer with a highly heterogeneous clinical behavioral pattern (1, 2) due to its origin in neuroblasts, cells that are part of the neural crest which arises from the ectoderm of the embryo (3). The cells of the neural crest can differentiate into a wide range of cell types, including neurons, glia and melanocytes. Historically, neural crest cells beyond the post-migrational stage were thought to no longer retain multipotent properties, but current data indicates subpopulations are able to retain high plasticity (2–6). This leads to the formation of neuroblast-origin tumors, in the adrenal, medulla, skin and kidneys (3). Another outcome of the highly heterogenous nature of NB is the existence of three distinct cell subtypes: N-type neuroblastic/neuroendocrine precursors, S-type Schwannian/melanoblastic precursors and I-type intermediates. Each of these subtypes express distinct behaviors and morphologies. I-type cells share properties of both N- and S-type cells (2, 7, 8) and also retain the capability of stem cells to grow as mixtures of other cell types and the ability to develop into either of them. N-type cells have a neuronal morphology and are very invasive. S-type cells are substrate adherent, non-invasive and share a resemblance to glial precursor cells (7–9). NB manifests as two discrete subcategories: adrenergic and mesenchymal cell types. Epigenetic and transcriptomic profiling have been used to identify adrenergic and mesenchymal cell types (10). Upregulated expression of the paired-like homeobox 2 (PHOX2B) gene, the tyrosine hydroxylase (TH) gene and the dopamine beta-hydroxylase (DBH) gene are characteristic of adrenergic NB while the distinguishing epigenetic features of mesenchymal NB are an increased expression of vimentin or fibronectin (10). Recent single-cell analyses have also assisted in NB characterization from which a link between less differentiated chromaffin or symphoblast cell types, including nascent cells of neural crest development, and mesenchymal-like features, such as Schwann cell precursors, have been observed (11, 12). It should be noted that mesenchymal/neural crest cells are generally more resistant to conventional treatment methods such as chemotherapy and radiation therapy (12).

NB prognosis is correlated with the degree of cell differentiation as well as patient age. This leads to variable survival rates among patients from relapse and mortality to high survival rates, including tumor regression (13). Standard therapeutics for the disease are chemotherapy, radiation and surgical resection. However, as with other cancers, more aggressive NB may become resistant to these therapies. For this reason, research of new and novel therapeutics are necessary; these treatments focus on a more specific mode of operation by targeting molecular mechanisms that regulate processes such as apoptosis, angiogenesis, and metastasis (13–17).

The protein kinase C (PKC) family contains 16 isoforms belonging to one of three classes, conventional, novel, or atypical, based on the secondary messenger requirements. Atypical PKCs (aPKCs) contain two isoforms: PKC-iota (PKC-ι) and PKC-zeta (PKC-ζ). Both aPKCs are reported to have pro-oncogenic behaviors in many cancers (18–20). PKC-ι promotes cell proliferation in neuroblastoma cells through the PKC-ι/cyclin dependent kinase (Cdk7)/Cdk2 pathway (17). Overexpression of PKC-ζ has been linked to the development of malignancies such as prostate, ovarian, lymphoma, breast, melanoma, and bladder cancer (19, 21–32). We have previously shown that both PKC-ι and PKC-ζ induced prostate cell survival by promoting NF-κB activation (16, 30, 31). In addition, both aPKCs activate Vimentin to facilitate prostate cell motility and invasion (16, 32). PKC-ζ was found to protect cancer cells in the presence of the cytotoxic agent, Etoposide (VP-16) (33). Both aPKCs stimulate cell proliferation and differentiation of ovarian and melanoma cells (24, 27–30, 34). Higher levels of PKC-ι are detected in several tumors, including pancreas, lung, colon, breast, prostate, ovarian and melanoma (35, 36). We previously reported that *c-Jun* induced PKC-ι, which is largely responsible for promoting epithelial-mesenchymal transition (EMT) in melanoma and prostate carcinoma (23, 27, 35). The *PRKCI* gene is amplified in various carcinomas, even if PKC-ι overexpression is not always associated with gene amplification (37–39).

Human 14-3-3 proteins include seven isoforms (β, γ, ϵ, η, σ, τ, and ζ) of phospho-binding proteins that control nearly all key cellular functions. These connections make 14-3-3 proteins a crucial component of the signaling network that controls essential cancer processes such as apoptosis, autophagy, cell cycle progression, motility, and glucose metabolism (37, 38). 14-3-3 proteins function as either hetero- or homodimers. 14-3-3s may act as molecular adapters that link other proteins together due to their ability to alter the structure of the binding partner (38, 39). The *YWHAZ* gene (14-3-3ζ) promotes chemoresistance that is often associated with poor patient outcomes. *YWHAZ* locates in the 8q22.3 chromosomal region, which is frequently duplicated in cancers (37, 39). Many studies report 14-3-3ζ works as a central point to promote oncogenic and chemoresistance pathways in cancers such as PI3K/Akt1, Erk/Mapk and TGF-β (40). Gunaratne et al. suggested that aPKCs upregulate TGF- β pathway through the association with Smads (41). Warner et al. showed that Par3/Par6/aPKC complex interacts with Cdc42, Rac1 and Smads in relation to controlling cell polarity of dividing neuroblasts (42). Hurd et al. also suggested that aPKCs interact with 14-3-3 *via* Par3/Par6/aPKC complex to regulate cell polarity (43). Yang et al. showed that PKC-ι interacts with 14-3-3 to upregulate EMT in cholangiocarcinoma (44). In addition, 14-3-3 facilitates the interaction between PKC-ζ and Rac1 (45). Both PKC-ι and PKC-ζ are thought to undergo the maturation process *via* protein-protein interactions (46–48). Phosphoinositide-dependent kinase-1 (PDK1) is an important kinase during this process and 14-3-3ζ is known to mediate the signal propagation between PDK1 and Akt1 (46). In addition, we previously reported the role of aPKCs in upregulation of cancer progression in melanoma and prostate cancer *via* the TGF-β pathway (16, 28). Freeman and Morrison discuss the 14-3-3’s ability to bind many signaling molecules, thereby participate in wide array of cellular activities (49). Here, we hypothesize that aPKCs are essential for NB progression while 14-3-3 and Smad2/3 play central roles by coordinating aPKCs in the cell cycle (via the aPKC/Cdk7/Cdk2 pathway) and cancer progression *via* Akt1/NFκB/TGF-β pathways.

In the current study, we report the effects of aPKC inhibition on 14-3-3-centered signaling in NB progression. We demonstrate the downstream effects of aPKC attenuation on aPKC phosphorylation, the Akt1/NF-κB pathway, the Cdk7/Cdk2 pathway and EMT in relation to 14-3-3 and Smad2/3. We have analyzed the expression of EMT markers including Vimentin, a mesenchymal marker that is a hallmark of cancer metastasis. Data suggest that aPKC mitigation significantly downregulated 14-3-3/Smad signaling. Overall, our results suggest that both aPKCs are crucial for NB progression and metastasis along with 14-3-3 proteins. Results also indicate that aPKC attenuation retards the action of 14-3-3, thereby providing a novel potential therapeutic pathway for NB.

## Materials and methods

### Materials

5-amino-1-2,3-dihydroxy-4-(methylcyclopentyl)-1H-imidazole-4-carboxamide (ICA-1S) was supplied by Therachem (Jaipur, India) and 8-hydroxy-1,3,6-naphthalenetrisulfonic acid (ζ-Stat) (NSC 37044) was provided by the National Institute of Health (NIH, Bethesda, MD, USA). Sterile distilled water was used as the solvent. Materials were acquired as follows. Primary antibodies of PKC-ζ (sc-17781, Santa Cruz Biotech), PKC-ι (610175, BD Biosciences), p-PKC-ι (T555, ab5813), p-Cdk7 (T170, ab155976, Abcam), p-PKC-ζ (T410, PA5-78127, Invitrogen) and E-Cadherin (701134, Thermo Fisher Scientific). From Cell Signaling Biotechnology: Akt1 (4691S), p-Akt1 (S473, 4060S), p-Akt1 (T308, 13038S), p-IκBβ (T19/S23, 4921S), IκB (4814S), p-IKKα/β (S176/180, 2967S), IKKα (2682S), p-NF-κB p65 (S536, 3033S), NF-κB p65 (8242S), p-PTEN (S380, 9551S), PTEN (9188S), 14-3-3 (8312S), p-14-3-3 (9601S), Vimentin (5741S), p-Vimentin (S39, 13614S), p-Vimentin (S56, 7391S), N-cadherin (13116S), p-Smad2 (S465/467)/Smad3 (S423/425) (8828S), PARP (9532S), cleaved-PARP (5625S), Caspase-3 (9692S), cleaved-Caspase-3 (9664S), Bcl-2 (5071S), Bim (2933S), TRAIL (3219S), Puma (98672S), Cdk7 (2916S), Cdk2 (18048S) and p-Cdk2 (T160, 2561S). From Enzo Life Sciences: p-Vimentin (S6, ADI-KAM-CC245-E) and p-Vimentin (S33, ADI-KAM-CC246-E). β-actin-peroxidase (A3854) was obtained from Sigma. From OriGene: *si*RNA (human small interfering RNA) for PKC-ζ (SR321432), PKC-ι (SR321426). From Sigma Aldrich: DPBS without Mg^2+^ and Ca^2+^ ions (Dulbecco’s phosphate buffered saline, D8537) and Trypsin–EDTA (Ethylenediaminetetraacetic acid, T4049). Enhanced chemiluminescence (Super Signal West Pico Chemiluminescent Substrate) (34580) was purchased from Pierce (Rockford, IL). Horseradish peroxidase (HRP) conjugated goat anti-mouse (1706516), and goat anti-rabbit (1706515) secondary antibodies were obtained from Bio-Rad Laboratories (Hercules, CA). Matrigel basement membrane matrix (Corning 356234) and Transwell plates (Corning 3464) were purchased from Thermo Fisher Scientific.

### Cell culture

Human BE(2)-C (ATCC^®^ CRL-2268^™^), BE(2)-M17 (ATCC^®^ CRL-2267^™^) and human embryonic kidney (HEK)-293 (ATCC^®^ CRL-1573^™^) cells were purchased from the American Tissue Type Collection (ATCC, Rockville, MD). All cell lines were authenticated by ATCC using karyotyping, morphology, and PCR-based approaches. Early passages of cells were cryo-preserved in liquid nitrogen and cells of early passages were resuscitated from liquid nitrogen for experiments. Cell culture conditions were maintained at 37°C and 5% CO_2_. A 1:1 mixture of Eagle’s minimum essential medium (EMEM) (ATCC 30-2003) and F-12K medium (ATCC 30-2004) was used with fetal bovine serum (FBS, 10% v/v) and penicillin (5 µg/ml) for both NB cell lines; EMEM with 10% v/v FBS and penicillin (5 µg/ml) was used for HEK-293 cells.

### Inhibitor dose response curves for cell viability

HEK-293, BE(2)-C and BE(2)-M17 cells (4 × 10^4^) were cultured in T25 flasks and treated with either an equal volume of sterile water (vehicle control) or inhibitors (ICA-1S and ζ-Stat) using a series of concentrations (10, 25, 50, 75, and 100 μM) over a 3 day period. Combination treatments (12.5 + 20 µM, 25 + 37.5 µM, 50 + 75 µM of ICA-1S and ζ-Stat) were also conducted. The procedure was performed as detailed in Ratnayake, et al. (35).

#### Wound healing assay

The wound healing assay for BE(2)-C and BE(2)-M17 cells as described in Justus, et al. (50) was followed. Cells were treated with either sterile water (vehicle control) or inhibitors to achieve respective half maximal inhibitory concentrations (IC_50_) and plates were incubated at 37°C and 5% CO_2_. These IC_50_ values were obtained based on the dose response curves. Photographs of wound closure were taken using a Motic AE31E microscope with Moticam BTU8 Tablet (40x magnification) at 24 h intervals for 2 days and analyzed using the “ImageJ” image processing program (NIH, Rockville, MD, USA).

##### Cell migration and invasion assay

*In-vitro* migration/invasion assay was performed for both NB cell lines as described in protocol (https://www.corning.com/catalog/cls/documents/application-notes/CLS-AN-432_DL1.pdf). Cells were treated with either sterile water or inhibitors to achieve respective IC_50_ concentrations and plates were incubated at 37°C and 5% CO_2_ for 3 days. At the end of the 3rd day, crystal violet (0.5%) was used to stain the cells adhered to the bottom surface of the membrane in transwell insert. Photographs of the stained cells were taken from Motic AE31E microscope (40 × magnification). Subsequently, 70% Ethanol (200 μl) was added to dissolve crystal violet and absorbance was measured at 595 nm.

### Western blot analysis and densitometry

Approximately 1 × 10^5^ cells (BE(2)-C and BE(2)-M17) were cultured in T75 flasks; 24 h post-plating, fresh media were supplied, and cells were treated with either sterile water (vehicle control) or aPKC inhibitors at IC_50_ values. Additional doses were applied every 24 h during a 3-day incubation period. Cells were subsequently lifted, and cell lysate collected with cell lysis buffer (C7027, Invitrogen). Samples were then fractionated by sodium dodecyl sulfate–polyacrylamide gel electrophoresis (SDS-PAGE) and immunoblotted. Western blots were done following the procedure given in Ratnayake, et al. (35). Western blot band intensities were calculated using the software “AlphaView” (ProteinSimple Inc., San Jose, CA, USA) to estimate corrected protein intensity by subtracting the baseline intensity from each band.

### Knockdown of PKC-ι and PKC-ζ expression

BE(2)-C and BE(2)-M17 (1 × 10^5^) cells were seeded in T75 flasks and 24 h post-seeding, fresh media were supplied and short interfering RNA (*si*RNA) (30 nM for PKC-ι/ζ) treatments were conducted against scrambled *si*RNA (control) for 48 h using ‘*si*Tran’ siRNA transfection reagent (TT300002, Origene Technologies, Inc.) according to the manufacturer’s recommended ratios. Cell pellets were collected at the end of the 48 h incubation period for Western blot or qPCR experiments, as described in Win, et al. (24). Duplex sequences used in *si*RNAs were, for PKC-ι: rGrUrArUrUrCrArCrUrUrCrArArArUrCrArUrArArArCrUTA and for PKC-ζ: rGrArGrGrArArUrArArArArUrGrUrUrCrCrGrArUrGrUrUGT.

### Flow cytometry; cell cycle analysis

BE(2)-C and BE(2)-M17, approximately 1 × 10^5^ cells, were cultured in T75 flasks; 24 h post-plating, fresh media were supplied, and cells were treated with either sterile water (vehicle control) or aPKC inhibitors at IC_50_ values. Additional doses were applied every 24 h during a 3-day incubation period. Cells were subsequently lifted and washed in PBS. Cells were then fixed in cold 70% ethanol added drop wise to the pellet while vertexing. Cells were allowed to fix 30 min at 4°C.with gentle rocking. Samples were then washed 2X in cold PBS. Subsequently they were spun at 850 g in a centrifuge. Following this the cells were treated the cells with 50 µl of a 100 µg/ml stock of RNase, then 200 µl Propidium iodide (from 50 µg/ml stock solution) was added and then cells were filtered through a ~ 70 µm nylon filter and analyzed on a flow cytometer.

### Immunofluorescence microscopy

Slides were prepared for BE(2)-C and BE(2)-M17 cells as previously described in Ratnayake, et al. (35) for aPKC specific inhibitor treatments (ICA-1S and ζ-Stat). At the end of day 2 of treatment, cells were stained for either PKC-ι or PKC-ζ (red), along with Smad2/3 (yellow) and 14-3-3 (green) for visualization under a Leica DM2000 upright fluorescent microscope using a Spot Flex cooled CCD Camera (Leica Microsystems Inc., Buffalo Grove, IL) (63x magnification). Images were captured and analyzed using “Spot Basic” software (SPOT Imaging, Sterling Heights, MI). 4′,6-diamidino-2-phenylindole (DAPI) staining was used to visualize the nucleus (S36938; blue).

### Immunoprecipitation

PKC-ι, PKC-ζ and 14-3-3 were separately immunoprecipitated (IP) from 500 μg total protein sample. Agarose conjugated PKC-ι, PKC-ζ and 14-3-3 primary antibodies were used, and the manufacturer’s protocol was followed to immunoprecipitate the protein. Precipitated proteins were separated by SDS-PAGE and analyzed using Western blot techniques to determine the associated proteins with either PKC-ι, PKC-ζ, 14-3-3 and Smad2/3.

### Statistical analysis

All data are presented as mean ± SEM. Statistical analysis was performed with a one or two-way ANOVA followed by Tukey’s HSD test as multiple comparisons tests using the GraphPad Prism software for statistical analysis. A *P*-value of less than or equal to 0.05 indicated statistical significance.

## Results

### Dose response curves demonstrate ICA-1S and ζ-Stat significantly affect NB cell lines

**Figure 1:**
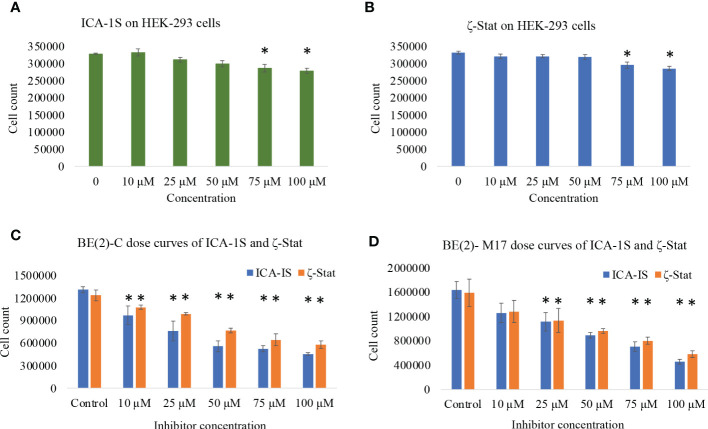
The effects of aPKC inhibitors (ICA-1S and ζ-Stat) on cell proliferation, cell viability and cytotoxicity for HEK-293, BE(2)-C and BE(2)-M17 cells. Dose response curves showing **(A)** effect of ICA-1S on HEK-293; **(B)** effect of ζ-Stat on HEK-293; **(C)** effect of ICA-1S/ζ-Stat on BE(2)-C; and **(D)** effect of ICA-1S/ζ-Stat on BE(2)-M17. Mean ± SD (*N* = 3) are plotted. Statistical significance is indicated by asterisk as *P <0.05.

We analyzed the downstream effects of inhibition of PKC-ι using PKC-ι specific inhibitor ICA-1S and PKC-ζ using PKC-ζ specific inhibitor ζ-Stat. ICA-1S decreased HEK-293 proliferation by 12.2% at 100 µM (*P* ≤0.05) while ζ-Stat decreased growth by 15.1% at 100 µM (*P* ≤0.05) (**Figures 1A, B**). Both inhibitors decreased cell proliferation of BE(2)-C and BE(2)-M17 cells significantly more compared to HEK-293. ICA-1S decreased proliferation of BE(2)-C cells by 26.0% for 10 µM, 41.9% for 25 µM, 57.4% for 50 µM and 65.4% for 100 µM (all *P* ≤0.05) and in BE(2)-M17 cells by 23.0% for 10 µM, 31.9% for 25 µM, 45.5% for 50 µM and 72.1% for 100 µM (all *P* ≤0.05) (**Figure 1C**). ζ-Stat decreased proliferation in BE(2)-C cells by 12.8% for 10 µM, 19.9% for 25 µM, 37.8% for 50 µM, 47.9% for 75 µM and 53% for 100 µM (all *P* ≤0.05) while in BE(2)-M17 cells, growth was reduced by 19.2% for 10 µM, 28.3% for 25 µM, 41.3% for 50 µM, 49.5% for 75 µM and 66.4% for 100 µM (*P* ≤0.05) (**Figure 1D**). This suggests that these inhibitors can effectively decrease cell population size in NB cells. IC_50_ values of ICA-1S and ζ-Stat were approximately 50 µM and 75 μM, respectively, for both cell lines. We thus used these concentrations as testing concentrations in subsequent experiments. Combinational doses of both inhibitors were less effective in decreasing cell proliferation of BE(2)-C and BE(2)-M17 cells compared to individual doses. In BE(2)-C combinational treatments, 12.5 + 20 µM and 25 + 37.5 µM of ICA-1S and ζ-Stat respectively, displayed no significant effect in decreasing cell proliferation (*P* ≤0.05) compared to individual doses in both cell lines (**Supplementary Figure 1**). Only 50 + 75 µM of ICA-1S and ζ-Stat demonstrated a significant decrease in cell population in both cell lines (*P* ≤0.05), but the degree of cell population reduction is much greater in individual doses compared to combination trials. This suggests that these inhibitors lack synergistic effect and are less effective at decreasing cell population size in NB cells in combination.

### Inhibitors of aPKC expression caused cell cycle arrest and induced apoptosis

**Figure 2:**
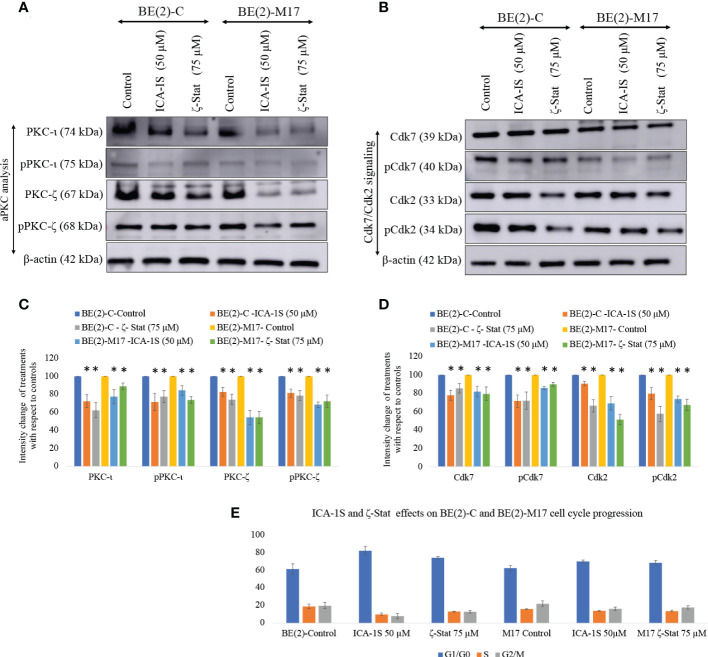
Effect of ICA-1S and ζ-Stat on aPKC expression and cell cycle in NB cells. **(A)** Western blots for protein expression levels of total PKC-ι, phosphorylated PKC-ι (T555), total PKC-ζ and phosphorylated PKC-ζ (T410). **(B)** Western blots for expression of Cdk7, phosphorylated Cdk7, Cdk2 and phosphorylated Cdk2 for the inhibitor treatments (50 µM of ICA-1S and 75 μM of ζ-Stat). Protein (60-80 µg) was loaded in to each well and β-actin was used as the housekeeping marker in each Western blot. **(C)** Densitometry bar graph for Western blots in A). **(D)** Densitometry bar graph for Western blots in B). **(E)** Flow cytometric cell cycle analysis demonstrates the percentage of different cell cycle phases on NB cells for ICA-1S and ζ-Stat treatments. Experiments (*N* = 3) were performed and representative bands are shown. Graphs depict percentage change of treated samples with respect to their controls and mean ± SD are plotted. Statistical significance is indicated by asterisk as *P < 0.05.

Western blots revealed that both inhibitors significantly reduced total and phosphorylated PKC-ι. ICA-1S (50 µM) reduced the expression of PKC-ι by 27.7% (*P* ≤0.05) and 32.0% (*P* ≤0.05) in BE(2)-C and BE(2)-M17, respectively. Interestingly, ζ-Stat (75 µM) also reduced the expression of PKC-ι by 37.6% (*P* ≤0.05) and 28.0% (*P* ≤0.05) in BE(2)-C and BE(2)-M17, respectively. Similarly, pPKC-ι (T555) was reduced by 15.2% (*P* ≤0.05) and 44.6% (*P* ≤0.05) in BE(2)-C and BE(2)-M17, respectively by ICA-1S while ζ-Stat (75 µM) reduced pPKC-ι by 22.0% (*P* ≤0.05) and 49.5% (*P* ≤0.05) in BE(2)-C and BE(2)-M17, respectively (**Figures 2A–D**).

Both ICA-1S and ζ-Stat treatments also led to a significant decrease in total and phosphorylated levels of PKC-ζ. ICA-1S (50 µM) reduced the expression of PKC-ζ by 14.2% (*P* ≤0.05) and 42.0% (*P* ≤0.05) in BE(2)-C and BE(2)-M17, respectively. ζ-Stat (75 µM) reduced the expression of PKC-ζ by 23.3% (*P* ≤0.05) and 45.6% (*P* ≤0.05) in BE(2)-C and BE(2)-M17, respectively. Similarly, pPKC-ζ (T410) was reduced by 20.6% (*P* ≤0.05) and 25.4% (*P* ≤0.05) in BE(2)-C and BE(2)-M17, respectively by ICA-1S while ζ-Stat reduced pPKC-ζ (T410) by 48.8% (*P* ≤0.05) and 50.4% (*P* ≤0.05) in BE(2)-C and BE(2)-M17, respectively (**Figures 2A–D**). We have also analyzed some selected markers for combination treatments as shown in **Supplementary Figure 1** which showed lesser effects of combination treatments on aPKCs and related pathways compared to individual treatments of ICA-1S and ζ-Stat.

Both inhibitors can also induce cell cycle arrest in NB cells. Both ICA-1S and ζ-Stat significantly reduced the levels of Cdk7, pCdk7, Cdk2 and pCdk2 compared to controls in both cell lines, indicating cell cycle arrest (**Figures 2B, D**). Flow cytometric analysis of cell cycle demonstrated that 61.2% cells were in G1/G0 in BE(2)-C cells control compared to 82.2% and 74.1% in ICA-1S and ζ-Stat treatments, respectively (**Figure 2E**). In addition, the percentages of the cells in S phase were 19.0%, 9.9% and 13.1% for BE(2)-C control, ICA-1S and ζ-Stat treatments, respectively. Percentages of the cells in G2/M phase were 19.8%, 7.9% and 12.8% for BE(2)-C control, ICA-1S and ζ-Stat treatments, respectively. (**Figure 2E**). Similarly, 62.2% cells were in G1/G0 in BE(2)-M17 cells control compared to 70.0% and 68.8% in ICA-1S and ζ-Stat treatments, respectively (**Figure 2E**). In addition, the percentages of the cells in S phase were 15.8%, 13.8% and 13.7% for BE(2)-M17 control, ICA-1S and ζ-Stat treatments, respectively (**Figure 2E**). Percentages of the cells in G2/M phase for BE(2)-M17 cells were 22.0%, 16.2% and 17.8% for the control, ICA-1S and ζ-Stat treatments, respectively (**Figure 2E**). These results further confirmed that the aPKC specific inhibition using ICA-1S and ζ-Stat induce cell cycle arrest in NB cells.

**Figure 3:**
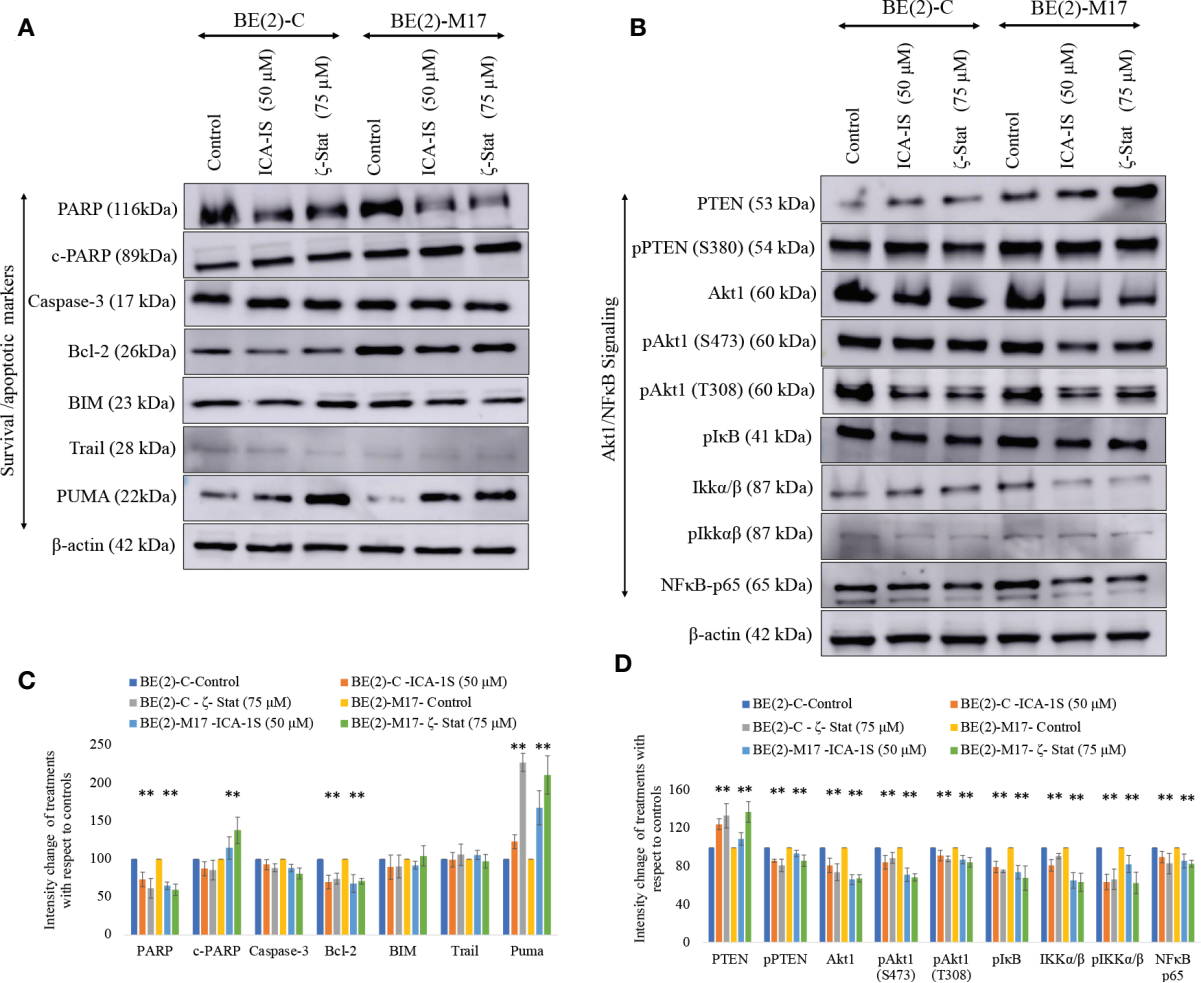
Effect of ICA-1S and ζ-Stat on apoptosis and Akt1/NF-κB signaling in NB cells. **(A)** Western blots of the expression of the protein levels of total PARP, cleaved PARP, Caspase-3, cleaved Caspase-3, Bcl-2, BIM, TRAIL and PUMA. **(B)** Western blots of the expression of PTEN, phosphorylated PTEN, Akt1, phosphorylated Akt1 (S473), phosphorylated Akt1 (T308), phosphorylated IκB, IKKα/β, phosphorylated IKKα/β and NF-κB p65 for the inhibitor treatments (50 µM of ICA-1S and 75 μM of ζ-Stat). Protein (60-80 µg) was loaded into each well and β-actin was used as the housekeeping marker in each Western blot. **(C)** Densitometry bar graph for Western blots in A). **(D)** Densitometry bar graph for Western blots in B). Experiments (*N* = 3) were performed and representative bands are shown. Graphs depict percentage change of treated samples with respect to their controls and mean ± SD are plotted. Statistical significance is indicated by asterisk as *P < 0.05.

Since both inhibitors significantly reduce NB cell proliferation, we tested the potential of inhibitors to induce apoptosis in NB cells. Cleaved-PARP and PUMA levels were all significantly increased while pro-survival Bcl-2 and total PARP levels were significantly decreased by inhibitor treatments (**Figures 3A, C**). Increased PARP cleavage and PUMA upregulation indicate induced apoptosis. Pro-apoptotic Caspase-3, BIM and TRAIL levels were also tested but levels were not altered significantly. These changes indicate that induced apoptosis takes place in NB cells upon aPKC inhibition from ICA-1S and ζ-Stat treatments.

### aPKC inhibition leads to downregulation of Akt1/NF-κB signaling

aPKC-ι/ζ inhibition with ICA-1S and ζ-Stat decreased total Akt1, phosphorylated Akt1 (S473), phosphorylated Akt1 (T308), total NF-κB p65, phosphorylated PTEN (S380), phosphorylated IκBα and phosphorylated IKKα/β (S176/180) (**Figures 3B, C**). In contrast, a significant increase in total PTEN occurred. Therefore, the data suggests that PTEN is stabilized and an overall downregulation of Akt1/NF-κB signaling.

### Migration and invasion assays show aPKC inhibition decreases NB cell migration/invasion characteristics

**Figure 4:**
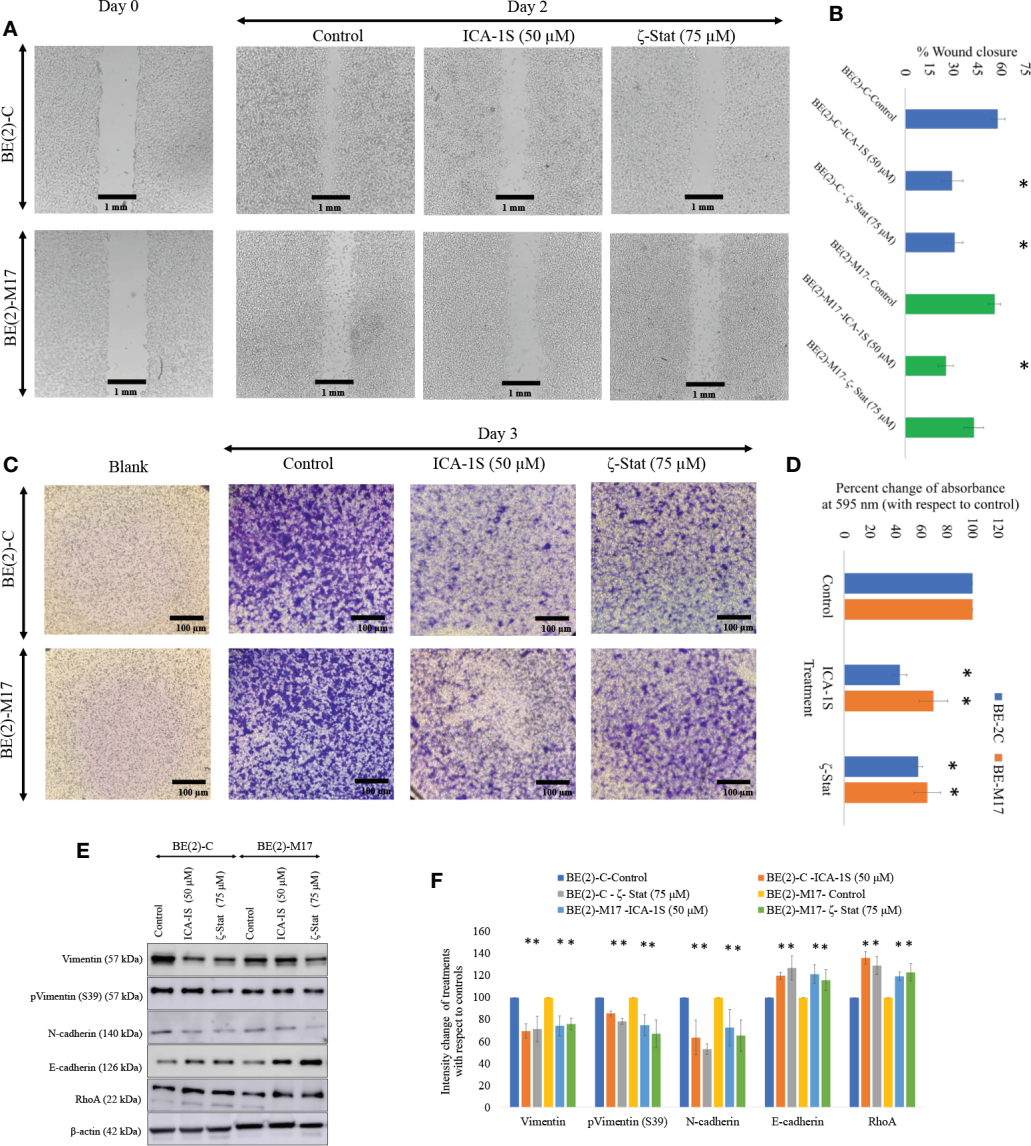
Inhibitors decrease NB cellular migration and invasion. **(A)** The effect of aPKC inhibitors (50 µM of ICA-1S and 75 μM of ζ-Stat) on NB cell migration in the wound healing assay. Microscopic photographs (40x) of scratches on cells at the beginning (day 0) were compared with the photographs taken after 2 days for BE(2)-C and BE(2)-M17 cells. **(B)** A comparison of calculated percent wound closure for the photographs taken using ImageJ (NIH, Rockville, MD, USA). Experiments (*N* = 3) were performed for each cell line and representative photographs are shown. **(C)** Representative photographs of invaded cells in the bottom surface of transwell were stained with 0.5% crystal violet and microscopic photographs were taken (40×). **(D)** Subsequently, crystal violet was dissolved in 70% ethanol and absorbance was measured at 595 nm which is directly proportional to the number of invaded cells. **(E)** Western blots of the expression of the protein levels of total Vimentin, phosphorylated Vimentin (S39), N-cadherin, E-cadherin and RhoA for the inhibitor treatments (50 µM of ICA-1S and 75 μM of ζ-Stat). Protein (60-80 µg) was loaded into each well and β-actin was used as the housekeeping marker in each Western blot. **(F)** Densitometry values for Western blots in E). Experiments (*N* = 3) were performed, and representative bands are shown. Graphs depict percentage change of treated samples with respect to their controls and mean ± SD are plotted. Statistical significance is indicated by asterisk as *P < 0.05.

Photographs of the wound closure were obtained (**Figure 4A**). Moreover, the areas of the scratch (wound) were measured and percentages of wound closure were calculated and compared to respective controls to measure the effects of inhibitor treatments (**Figure 4B**). In BE(2)-C control, wound closure was 57.6% (*P* ≤0.05) compared to wound closure levels of 29.2% (*P* ≤0.05) for ICA-1S (50 μM) and 30.8% (*P* ≤0.05) ζ-Stat (75 µM). Wound closure was 55.7% in the BE(2)-M17 control and 25.1% (*P* ≤0.05) for ICA-1S (50 μM) and 42.7% (*P* ≤0.05) for ζ-Stat (75 µM). Thus, both aPKC inhibitors significantly reduced NB cellular migration *in vitro* and ICA-1S was more effective in reducing cell migration compared to ζ-Stat. Moreover, Matrigel assay was executed using transwell inserts to examine the outcome of specific aPKC inhibition on *in-vitro* NB cellular migration/invasion. Invaded cells were treated with crystal violet on the transwell inserts and snapshots were captured as the visual representation of the invasion assay in randomly selected fields (**Figure 4C**). Crystal violet stained cells were then dissolved into the lower chamber in 70% ethanol and the absorbency was determined at 595 nm, which is directly proportional to the degree of invaded cells. (**Figure 4D**). These results suggested that ICA-1S decreased the cellular invasion by 56.7% (*P* < 0.05) and 42.4% (*P* < 0.05) for BE(2)-C and BE(2)-M17 cell lines, respectively. Similarly ζ-Stat decreased the cellular invasion by 30.5% (*P* < 0.05) and 35.3% (*P* < 0.05) for BE(2)-C and BE(2)-M17 cell lines, respectively.

Given the downregulating effects on cellular migration in the NB cells, we analyzed a few key EMT markers for ICA-1S and ζ-Stat treatments for NB cells (**Figures 4E, F**). Western blots and densitometries of EMT markers demonstrated that total Vimentin, phosphorylated Vimentin (S39) and N-cadherin protein levels were markedly reduced with inhibition of aPKCs while E-cadherin and RhoA were increased (**Figures 4E, F**). These results may indicate a slowing down of EMT in NB cells upon aPKC diminution.

*si*RNA for PKC-ι and PKC-ζ was initially tried and results were compared with the effects aPKC specific inhibitors to establish the specific inhibitory characteristics of ICA-1S and ζ-Stat. **Supplementary Figure 2** (Part B) demonstrates the effects of *si*RNA treatments on selected key markers and our preliminary data of *si*RNA for PKC-ι and PKC-ζ demonstrated that both 14-3-3 and Smad2/3 downregulated significantly more upon aPKC diminution compared to other tested markers. We thus analyzed the connection between aPKCs, 14-3-3 and Smad2/3 using Western blots and immunoprecipitation experiments, with an emphasis on the magnitude of changes observed using aPKC specific inhibitors.

**Figure 5:**
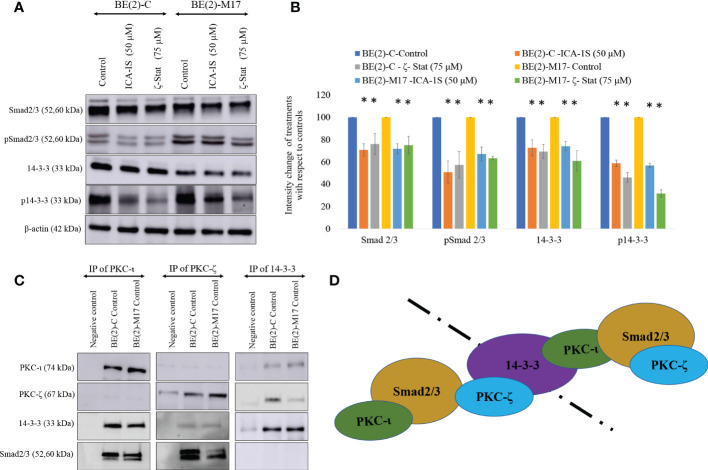
PKC-ι and PKC-ζ bind with 14-3-3 and Smad2/3. **(A)** Western blots of the expression of the protein levels of Smad2/3, phosphorylated Smad2/3, 14-3-3 and phosphorylated 14-3-3 for the inhibitor treatments (50 µM of ICA-1S and 75 μM of ζ-Stat). Protein (60-80 µg) was loaded into each well and β-actin was used as the housekeeping marker in each Western blot. **(B)** Densitometry values for Western blots in A) in which experiments (*N* = 3) were performed and representative bands are shown. Graphs depict percentage change of treated samples with respect to their controls and mean ± SD are plotted. **(C)** Whole cell lysates (100 µg) of BE(2)-C and BE(2)-M17 cells were immunoprecipitated separately for PKC-ι, PKC-ζ and 14-3-3 using specific beads. Western blots of PKC-ι, PKC-ζ, 14-3-3 and Smad2/3 were obtained for the immunoprecipitated samples. Experiments (*N* = 3) were performed. **(D)** Schematic representation of possible associations of the proteins as obtained from the data. Statistical significance is indicated by asterisk as *P < 0.05.

Western blots for aPKC specific inhibitors revealed that both inhibitors significantly reduced total and phosphorylated 14-3-3 and Smad2/3. ICA-1S (50 µM) reduced the expression of Smad2/3 by 29.3% (*P* ≤0.05) and 28.3% (*P* ≤0.05) in BE(2)-C and BE(2)-M17, respectively. ζ-Stat (75 µM) also reduced the expression of PKC-ι by 23.9% (*P* ≤0.05) and 24.8% (*P* ≤0.05) in BE(2)-C and BE(2)-M17, respectively. Interestingly, pSmad2/3 was reduced by 49.5% (*P* ≤0.05) and 32.8% (*P* ≤0.05) in BE(2)-C and BE(2)-M17, respectively by ICA-1S while ζ-Stat (75 µM) reduced pSmad2/3 by 42.5% (*P* ≤0.05) and 36.5% (*P* ≤0.05) in BE(2)-C and BE(2)-M17, respectively (**Figures 5A, B**).

Similarly, ICA-1S (50 µM) reduced the expression of 14-3-3 by 27.3% (*P* ≤0.05) and 25.9% (*P* ≤0.05) in BE(2)-C and BE(2)-M17, respectively. ζ-Stat (75 µM) also reduced the expression of 14-3-3 by 30.6% (*P* ≤0.05) and 38.9% (*P* ≤0.05) in BE(2)-C and BE(2)-M17, respectively. Interestingly, p14-3-3 was reduced by 42.2% (*P* ≤0.05) and 43.0% (*P* ≤0.05) in BE(2)-C and BE(2)-M17, respectively by ICA-1S while ζ-Stat (75 µM) reduced p14-3-3 by 53.9% (*P* ≤0.05) and 68.3% (*P* ≤0.05) in BE(2)-C and BE(2)-M17, respectively (**Figures 5A, B**).

### Immunoprecipitation and immunofluorescence microscopy suggest interactions of aPKCs with 14-3-3 and Smad2/3

Immunoprecipitation (IP) data indicate that PKC-ι and PKC-ζ associate with both 14-3-3 and Smad2/3. This was observed in both NB cell lines (**Figure 5C**). In addition, our IP experiments confirmed that no significant association exists between 14-3-3 and Smad2/3. Thus, it appears that both aPKCs communicate through 14-3-3 and Smad2/3. **Figure 5D** summarizes possible ways of aPKC association can occur with Smad2/3 and 14-3-3 based on our data. Both aPKCs demonstrate associations with Smad2/3 and 14-3-3 while no direct association is observed between 14-3-3 and Smad2/3 or between two aPKC isoforms.

**Figure 6:**
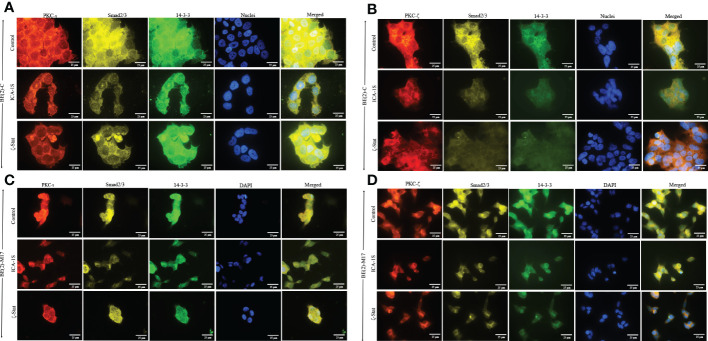
PKC-ι and PKC-ζ associations with 14-3-3 and Smad2/3 in NB cells. BE(2)-C and BE(2)-M17 (2.5×10^3^) cells were seeded in chamber slides and at 24 h post-seeding time, fresh media was supplied and inhibitor treatments (50 µM of ICA-1S and 75 μM of ζ-Stat) were applied every 24 h for 48 h. **(A)** Immunofluorescence staining of PKC-ι (red panel), Smad2/3 (yellow panel), 14-3-3 (green panel) and nuclei (blue panel), along with the merged image of all four colors, for BE(2)-C cells treated with either ICA-1S (50 µM) or ζ-Stat (75 µM) against its control. **(B)** Immunofluorescence staining of PKC-ζ (red panel), Smad2/3 (yellow panel), 14-3-3 (green panel) and nuclei (blue panel), along with the merged image of all four colors, for BE(2)-C cells treated with either ICA-1S (50 µM) or ζ-Stat (75 µM) against its control. **(C)** Immunofluorescence staining as in A) for BE(2)-M17 cells. **(D)** Immunofluorescence staining as in B) for BE(2)-M17 cells. Images were captured at 630x magnification and experiments (*N* = 3) were performed.

Immunofluorescence (IF) staining (**Figure 6**) confirmed the association of aPKCs with 14-3-3 and Smad2/3. Both cell lines demonstrated significant PKC-ι/ζ reduction upon treatment with ICA-1S and ζ-Stat, confirming downregulation of one aPKC member negatively affected the other aPKC member. Both 14-3-3 and Smad2/3 also decreased with inhibitor treatments, further confirming their association with PKC-ι/ζ.

## Discussion

We have previously discussed the specific inhibitory nature of ICA-1S and ζ-Stat against PKC-ι and PKC-ζ (31). Previously published molecular modeling and kinase activity data confirmed that ICA-1S inhibits the activity of PKC-ι without having a significant effect on PKC-ζ. Similarly, ζ-Stat inhibits PKC-ζ without having a significant effect on PKC-ι (31). In addition, these compounds did not demonstrate a significant effect on the total or phosphorylated levels of the other aPKC member when specifically inhibited with either ICA-1S or ζ-Stat in melanoma and prostate carcinoma. Given the ~70% similarity between the primary structures of PKC-ι and PKC-ζ catalytic domains, this was a key result to establish (27). In the current study, we show that specific inhibition of PKC-ι using ICA-1S led to a reduction of PKC-ζ levels in both NB cell lines and that PKC-ζ specific inhibition using ζ-Stat led to a reduction of PKC-ι levels in the NB cell lines. This result suggests that the aPKCs are dependent on each other, such that when one member is inhibited using a specific inhibitor, the other aPKC member is also downregulated. This may be due to crosstalk between the two aPKC members or through a direct association. Our IP data conformed that there is no direct association between the two aPKC isoforms but they demonstrated strong association with 14-3-3 and Smad2/3, which indicates that crosstalk between the aPKCs may occur through 14-3-3 and Smad2/3.

Quantifying the cytotoxicity of inhibitors against NB cells is key for establishing their therapeutic potential. Before applying ICA-1S and ζ-Stat on NB cells we tested their effects on HEK-293 cells. HEK-293 cells are often used as a control in studies involving the testing of treatment effects on cancer-specific cells. For example, these cells were recently used in this context in a study investigating the effect of thymoquinone on breast cancer (51). Both ICA-1S and ζ-Stat had only mild effects on the cell proliferation of HEK-293 cells. Also, neither compound had a significant effect on HEK-293 cell proliferation and viability at the IC_50_ concentrations (50 µM of ICA-1S and 75 µM of ζ-Stat) used for BE(2)-C and BE(2)-M17 cell lines. On the other hand, both inhibitors decreased the cell proliferation of NB cells at levels beyond 10 µM. ICA-1S displayed greater cytotoxicity on both cell lines compared to ζ-Stat. Nevertheless, both inhibitors were effective in arresting growth, differentiation and proliferation in BE(2)-C and BE(2)-M17 cells. Therefore, our data suggests that NB cells are highly dependent on aPKCs to remain viable.

Data suggested that total and phosphorylated protein levels of both Cdk7 and Cdk2 were moderately decreased as a result of ICA-1S and ζ-Stat treatment (**Figures 2B, D**). Cdk2 directs the transition from Gap1 phase to G1/S phase (DNA synthesis phase) while Cdk1 initiates mitosis. Cyclin-dependent kinase activating kinase (CAK) regulates these Cdks by acting as a central mediator of Cdk1/cyclin B and Cdk2/cyclin A complex formation and phosphorylation of the active segments (T loops) of both Cdk1 and Cdk2 (43, 44). Pillai, et al. suggested that Cdk7 and its downstream target Cdk2 are direct kinase targets of PKC-ι (17). They showed that PKC-ι directly associated with and phosphorylated both Cdk7 and Cdk2 thereby promoting NB cell proliferation (17). Current data demonstrate that both PKC-ι and PKC-ζ are equally responsible for upregulating the Cdk7/Cdk2 pathway in NB. This could also be due to crosstalk between the two aPKC members within the cellular environment. Pillai, et al. also noted that NB showed a 94-fold increase in PKC-ι in adrenal NB compared to normal adrenal biopsies (17). In addition, our preliminary data indicate that PKC-ι and PKC-ζ expression levels are higher in actively proliferating NB cells (25-80% confluent cells) than in 100% confluent cells (**Supplementary Figure 2A**).

Overexpression of aPKCs is associated with anti-apoptotic effects in many cancers. Our data show a reduction in total protein levels, as well as phosphorylated levels of aPKCs in ICA-1S and ζ-Stat treatments. As our data indicates, an increase in PUMA and PARP cleavage are pro-apoptotic downstream effects of aPKC inhibition of NB cells. In addition, decreases in Bcl-2 and total PARP levels indicate apoptosis stimulation upon inhibition of both PKC-ι and PKC-ζ (**Figures 3A, C**). Akt1 mediated NF-κB is one of the major anti-apoptotic pathways in which aPKCs play a role in releasing NF-κB to translocate to the nucleus and promote cell survival (31). PI3K stimulates IKKα/β through activation of Akt1 by phosphorylation at S473, which ultimately stimulates translocation of the NF-κB complex into the nucleus, boosting cell survival. PTEN regulates levels of PI3K. Phosphorylation at S380 leads to the inactivation of PTEN, thereby increasing the levels of PI3K followed by enhancement in phospho-Akt1 (S473) (31). Our data show that inhibition of PKC-ι and PKC-ζ significantly decrease the levels of phospho-PTEN and phospho-Akt1 while increasing PTEN levels in both NB cell lines (**Figures 3B, D**). This indicates that the PI3K/Akt1 activity is reduced because of aPKC inhibition. We also found that aPKC inhibition decreased the levels of phospho-IκB (S32) and phospho-IKKα/β (S176/180), confirming that both PKC-ι and PKC-ζ inhibition led to reduced Akt1 activation of IKKα/β. Increased levels of stabilized IκB (unphosphorylated IκB) therefore remain bound to the NF-κB complex, maintaining its inactive state and preventing NF-κB translocation to the nucleus to promote cell survival.

We also investigated the effects of aPKC inhibition on NB migration characteristics, with particular emphasis on EMT signaling. Downregulation of total Vimentin, phosphorylated Vimentin (S39) and N-cadherin and upregulation of E-cadherin and RhoA indicate a possible slowing down of EMT (32, 45). Downregulated aPKC expression reduced the motility of BE(2)-C and BE(2)-M17 cell lines. We also showed that corresponding Vimentin and phosphorylated Vimentin (S39) levels in both cell types were significantly decreased as a result of reduced aPKC expression (**Figures 4C, D**). These results demonstrate the acquisition of more epithelial characters while mesenchymal characteristics are reduced in NB cells *in vitro*. Our previous work has shown that when EMT slows down, downregulation of SNAIL1 and PRRX1 stabilizes E-cadherin mRNA expression resulting in constant E-cadherin transcription (19). On the other hand, E-cadherin stabilizes the tight junctions between cells. Upregulation of E-cadherin and downregulation of Vimentin expression helps cells gain apical-basal polarity which implies more epithelial characters (52–54).

**Figure 7:**
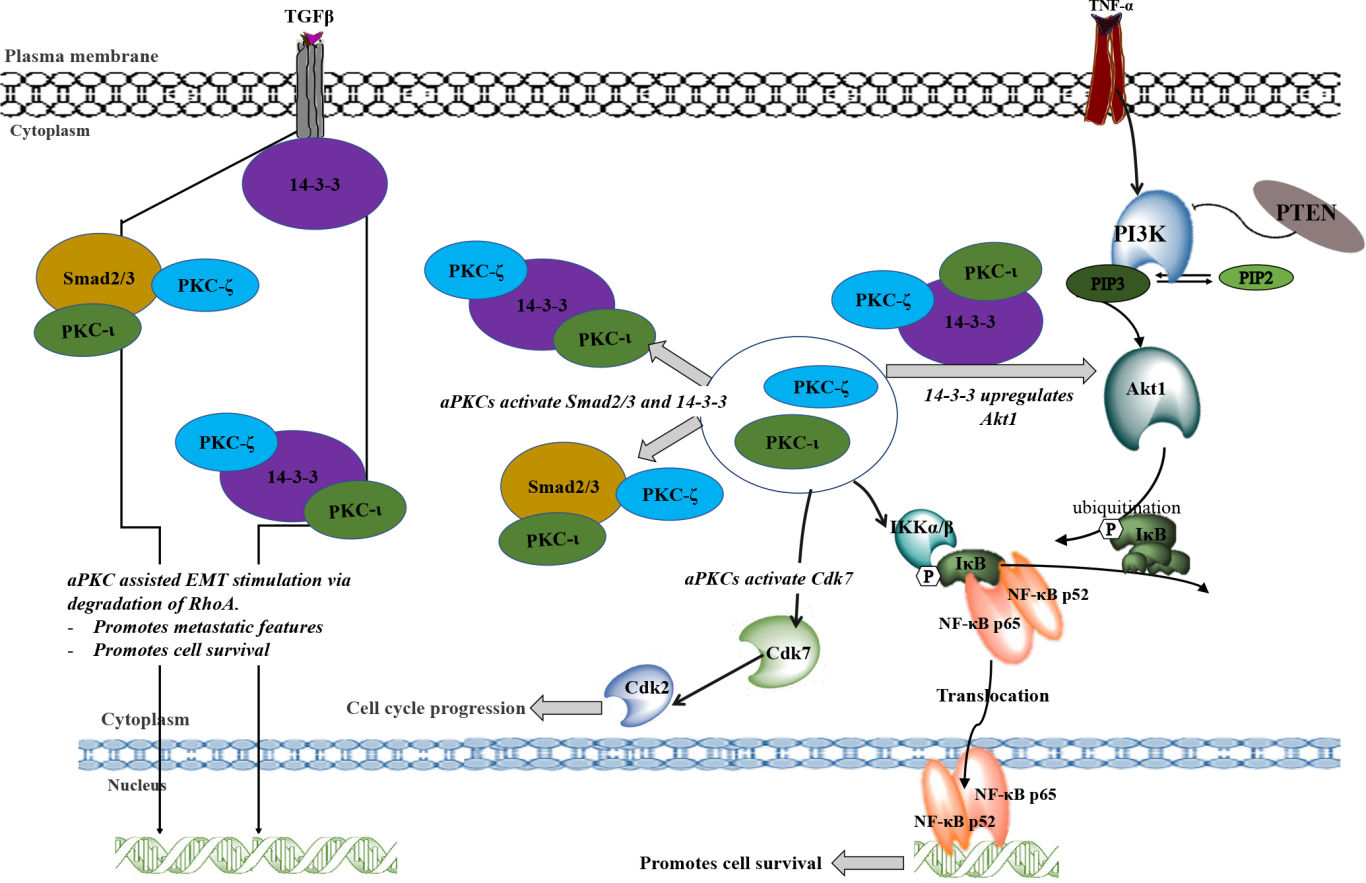
A schematic summary of the involvement of PKC-ι and PKC-ζ in NB progression. TNF-α and TGFβ may stimulate the aPKC activity which leads to associate with 14-3-3 and Smad2/3. These associations may upregulate key signaling points to upregulate Akt1/NF-κB pathway and the EMT stimulation by inducing the loss of E-cadherin and gain of Vimentin. During EMT stimulation, activated Smad2/3 and 14-3-3 (through the phosphorylation of aPKCs) will destabilize RhoA and induce transcription factors to induce EMT. On the other hand, activated 14-3- induces Akt1 which leads to the inactivation of inhibitory action of PTEN on PIP3. This may result in activation of Akt1 through the activated 14-3-3 *via* aPKCs. Activated Akt1 pathway also activates NF-κB pathway in addition to direct upregulation of activated aPKCs. Akt1/NF-κB pathways lead to cell survival, rapid proliferation, and differentiation of NB cells. In addition, activated aPKCs upregulate Cdk7 which leads to the cell cycle progression of NB cells. In summary, this schematic diagram shows how aPKCs are crucial for NB progression with respect to multiple cellular processes.

We further investigated EMT upon aPKC downregulation using additional markers. With aPKC inhibition, N-cadherin levels significantly decreased along with Vimentin, while RhoA increased along with E-cadherin. aPKC/Par6 have been shown to stimulate EMT when TGF-β receptors are activated (55). RhoA promotes stress-induced actin fiber formation and thus maintains epithelial cell integrity. TGF-β activation induces RhoA degradation, leading to de-polymerization of F-actin (filament actin), reducing structural integrity, and resulting in a decrease in the adhesion between cells (19, 56–65). In addition, TGF-β induces SNAIL, a crucial transcription factor that stimulates EMT *via* the Smad cascades (50, 51). TGF-β-induced activation of receptors leads to activation of Smad2 and Smad3 through direct phosphorylation by serine/threonine kinases in relation to 14-3-3. Phosphorylated Smad2 and Smad3 then form trimers with Smad4 and translocate into the nucleus, where they associate and cooperate with DNA binding transcription factors to activate or repress target gene transcription to facilitate EMT (56–59). As summarized in **Figure 7**, our data suggested that aPKCs are crucial mediators of the activities of 14-3-3 and Smad2/3 in EMT upregulation in NB cells. 14-3-3 may work as a central point to promote oncogenic and chemoresistance pathways in cancers such as PI3K/Akt, Erk/Mapk and TGF-β (40). The 14-3-3 protein family interacts with phosphorylated serine or threonine residues on target proteins, which are frequently within regions of intrinsic disorder and interact with the phospho-binding pocket on the 14-3-3 dimer (37, 60). The dynamic nature of 14-3-3 as phospho-binding adapter proteins, marks them as interesting candidates for a more thorough investigation into their role in aPKC regulation and their overall effect as modulators of multiple signaling pathways (37). Both PKC-ι and PKC-ζ are thought to undergo the maturation process *via* protein-protein interactions along with Pdk1 and Akt1, specifically with regard to PKC-ζ, which has been identified as a substrate of PDK-1 along with Akt1 (46). Therefore, we checked the levels of 14-3-3 and phosphorylated 14-3-3 in relation to Akt/NF-κB and Smad pathways. Our data suggest that aPKC mitigation significantly downregulates 14-3-3 and Smad2/3 signaling. As shown in **Figure 5**, 14-3-3 and Smad2/3 levels were significantly reduced in both NB cell lines as a result of aPKC inhibition.

Our IP and IF results suggest that both aPKCs actively participate in NB progression and that 14-3-3 and Smad2/3 act as coordinator molecules between PKC-ι and PKC-ζ. Both PKC-ι and PKC-ζ displayed direct associations with 14-3-3 and Smad2/3 but no significant direct associations were observed between PKC-ι and PKC-ζ or between 14-3-3 and Smad2/3. This suggests that both 14-3-3 and Smad2/3 act as central coordinators for crosstalk between PKC-ι and PKC-ζ in NB progression.

In summary, many of the core aPKC regulatory functions of NB cells could be regulated by association with, and consequent phosphorylation of, 14-3-3 and Smad2/3. In particular, TGFβ/Smad/aPKC pathway promotes EMT in BE(2)-C and BE(2)-M17 cells through direct coordination of 14-3-3, which is crucial to maintaining optimal levels of activated aPKCs. Both aPKCs are upregulated simultaneously as a result of 14-3-3 and Smad2/3 associations. Our data also indicate that the use of specific inhibitors such as ICA-1S and ζ-Stat not only decreases the survival of NB cells by downregulating Akt1/NFκB signaling, but also adversely affects cell cycle progression by down-regulating Cdk7/Cdk2/aPKC pathway. Finally, this newly modeled pathway can be used to develop more specific and effective therapeutics for advanced NB cancer patients based on PKC-ι/ζ therapy. The findings here highlight significant therapeutic implications which merit further research.

## Data availability statement

The original contributions presented in the study are included in the article/**Supplementary Material**. Further inquiries can be directed to the corresponding author.

## Author contributions

RW and A-DM conceptualization; BS, RW, LL and HR formal analysis; RW and A-DM investigation; BS, RW and A-DM methodology; RW and BS writing-original draft; BS, RW, LL and A-DM. writing-review and editing; A-DM resources; A-DM supervision; A-DM funding acquisition. All authors contributed to the article and approved the submitted version.

## Supplementary material

Supplementary Figure 1 Combinational treatments of ICA-1S and ζ-Stat demonstrate lack of synergistic effects on NB cell lines. Dose response curves showing **(A)** the effects of ICA-1S and ζ-Stat combination on BE(2)-C and **(B)** the effects of ICA-1S and ζ-Stat combination on BE(2)-M17. **(C)** Western blots showing the combinational effects of ICA-1S and ζ-Stat on aPKC expression and key markers 14-3-3 and Smad 2/3.

Supplementary Figure 2 Preliminary data on effects of confluency and siRNA on aPKC expression. **(A)** Western blots showing aPKC expression based on confluency in BE(2)-C and BE(2)-M17. **(B)** Western blots showing effects of siRNA knockdown of aPKCs on 14-3-3, Smad 2/3, Cdk7 and Cdk2.
