# Supplementary figures and images for: 14-3-3 and Smad2/3 are crucial mediators of atypical-PKCs: Implications for neuroblastoma progression
Source: Front Oncol. 2023 Jan 20;13:1051516. doi: 10.3389/fonc.2023.1051516 (PMC9910080; doi:10.3389/fonc.2023.1051516)

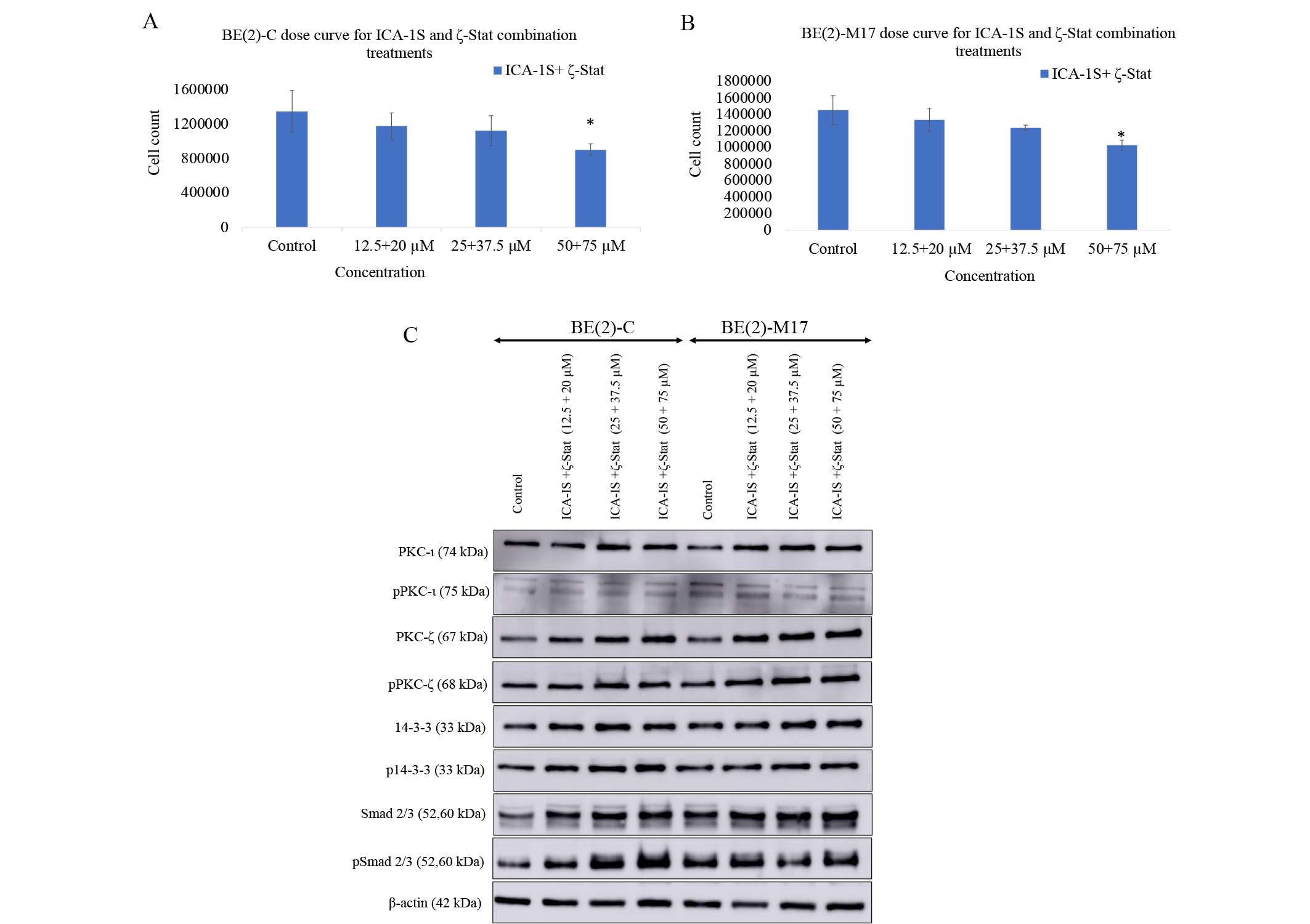

Supplement: Supplementary Figure 1 — Combinational treatments of ICA-1S and ζ-Stat demonstrate lack of synergistic effects on NB cell lines. Dose response curves showing (A) the effects of ICA-1S and ζ-Stat combination on BE(2)-C and (B) the effects of ICA-1S and ζ-Stat combination on BE(2)-M17. (C) Western blots showing the combinational effects of ICA-1S and ζ-Stat on aPKC expression and key markers 14-3-3 and Smad 2/3. [file Image_1.tif]

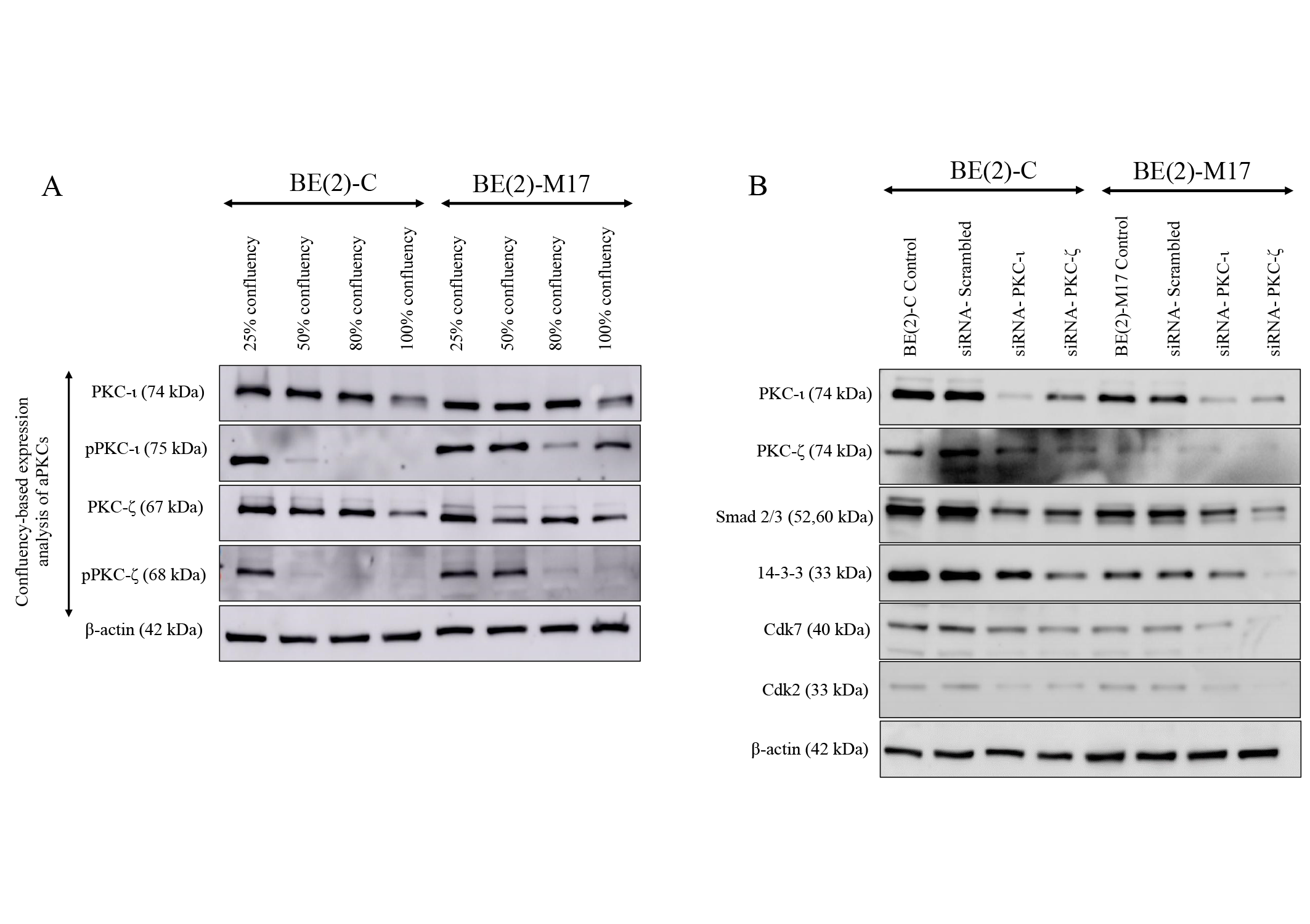

Supplement: Supplementary Figure 2 — Preliminary data on effects of confluency and siRNA on aPKC expression. (A) Western blots showing aPKC expression based on confluency in BE(2)-C and BE(2)-M17. (B) Western blots showing effects of siRNA knockdown of aPKCs on 14-3-3, Smad 2/3, Cdk7 and Cdk2. [file Image_2.tif]
